# Methods of Filling Teeth Forty Years Ago

**Published:** 1892-03

**Authors:** Frederick N. Seabury

**Affiliations:** Providence, R. I.


### METHODS OF FILLING TEETH FORTY YEARS AGO.[1]

BY FREDERICK N. SEABURY, D.D.S., PROVIDENCE, R. I.

Forty years ago dentists did not have cohesive gold, did not have rubber dam, did not have the mallet in any of its forms, nor the dental engine, nor at least a score of other instruments now deemed essential to properly equip an operative dentist. And yet teeth were filled then, and filled well. I believe the percentage of success was as high then as it is now.

When I started out from the Baltimore College, in the spring of 1849, I had an outfit of instruments of better quality and larger variety than the average student of that time ; but I could easily have tied the whole lot in my pocket-handkerchief.

Soft gold-foil was as well made as at present. Tin-foil was made, but the use of it was not considered respectable ; and as for amalgam, in the minds of a large majority of the leading dentists to use it was almost a crime. The feeling was that any tooth that could not be filled with gold had better be extracted.

It is not contemplated in this short paper to describe the different operations or treatment in vogue for preserving teeth at the time of which I speak, but to confine the paper and the discussion that may follow to the methods and some of the instruments used in filling teeth forty years ago.

---

[1] Read at the Twenty-fourth Annual Meeting of the American Academy of Dental Science, Boston, November 11, 1891.

In what I shall say I have in mind a simple cavity in the grinding surface of a molar, my object being to show the principle, and leave its application to the varying conditions as they are found in actual practice to your well-trained judgment. Contouring was only possible to a limited extent, as this system of filling required retaining walls.

In preparing a cavity, the rule was: If the depth of the cavity was equal to the diameter, the walls should be straight and parallel. In shallow and compound cavities the judgment of the operator must govern as to undercuts and retaining points.

In preparing the gold, two forms were in use. At the Baltimore College they rolled the gold into ropes, which were folded into the cavity, requiring that each loop should touch bottom, and stand out about one-fourth of its length. These loops were carried against the sides of the cavity with as much force as possible, great care being taken that no fold, after being placed in position, should be allowed to move. When no more folds could be carried to the bottom of the cavity, a wedge-shaped point was forced between them, and the gold carried each way against the walls. The hole thus made was plugged. This operation was repeated until the wedge-point could not be thrust in, when, with slightly serrated pluggers, the gold was pressed upon with all the strength the operator could exert, until it could be settled no more. Then with burnishers, hand-burs, and files the filling was cut back and finished.

The other method, and one taught me by my first preceptor, was to form the gold-foil into pellets; but the same principle governed. Each pellet was to touch bottom standing parallel, and leave sufficient gold standing out of the cavity to condense properly without being driven below its margin.

The difficulties in this kind of filling, which seems so simple in the description, are probably as great to a novice as any other. As I have said before, the gold must lie where it is first put, and on no account be moved in working in other pellets. The packing must be even all through, so that at the final condensing there shall not be hard and soft places in the filling. The amount of gold put in must be so calculated that the last piece may be carried below the surface, and condensed into the centre of the filling; for if the last piece comes out, or, from not being carried in, flakes off, you will have an imperfect filling, which will probably tumble out. But where these conditions are complied with, the filling may be condensed into a firm and practically solid mass, that will finish up well, and preserve the cavity from further decay. As a system of filling, it commends itself, first, for saving of time; second, less annoyance both to patient and operator; and, third, durability. I will agree to fill the cavity I have been considering, and finish it up, while another man is adjusting the rubber dam with the clamps and weights provided to keep it in position, and with very much less discomfort to the patient than is occasioned by all the paraphernalia made necessary to keep a cavity absolutely dry, so as to make a cohesive filling possible,—absolute dryness not being essential to the perfection of a wedged or soft gold filling.

There are plenty of fillings in the mouths of elderly people that have been doing good service for many years, that were put in, as we used to say, submarine; for if the gold is well put in, no man can tell the difference between the wet and dry, either in finish or durability. I sent to Dr. J. Foster Flagg a lower molar, with stopping on the buccal surface near the neck, that was filled by his father over fifty years before, in a position where I am sure he could not have kept it dry. The filling was in place, and the tooth perfectly preserved, it having dropped out from old age.

